# Long-term consequences of adolescent exposure to the synthetic cannabinoid AB-FUBINACA in male and female mice

**DOI:** 10.1016/j.isci.2025.111857

**Published:** 2025-01-20

**Authors:** Cristina Izquierdo-Luengo, María Ponce-Renilla, Marc Ten-Blanco, María Andrea Arnanz, Rosa María Tolón, Inmaculada Pereda-Pérez, Fernando Berrendero

**Affiliations:** 1 Institute of Life Sciences, Faculty of Experimental Sciences, Universidad Francisco de Vitoria, 28223 Pozuelo de Alarcón, Madrid, Spain; 2 Department of Psychobiology, Faculty of Psychology, Universidad Complutense, 28224 Pozuelo de Alarcón, Madrid, Spain

**Keywords:** natural sciences, biological sciences, neuroscience, behavioral neuroscience

## Abstract

The consumption of synthetic cannabinoids during adolescence is reported to be a risk factor for the appearance of psychiatric disorders later in life. AB-FUBINACA is a member of the indazole carboxamide family of synthetic cannabinoids present in Spice/K2 preparations. The present study sought to investigate the long-term effects of AB-FUBINACA consumption during adolescence in both male and female mice. AB-FUBINACA revealed several sex-dependent behavioral alterations. In this sense, the administration of this synthetic cannabinoid in female, but not male, mice induced psychotic-like symptoms which were associated with changes in dendritic arborization and density of mature dendritic spines in pyramidal neurons of the prefrontal cortex, as well as with an up-regulation of differentially expressed genes in this brain area. This study helps to clarify the potential late detrimental effects of this potent synthetic cannabinoid that may derive from its use during adolescence.

## Highlights

• Adolescents are more vulnerable to developing psychiatric disorders after SCB use
• AB-FUBINACA adolescent exposure alters memory and causes anxiety in adult female mice
• AB-FUBINACA adolescent exposure induces psychotic-like effects in adult female mice
• AB-FUBINACA adolescent exposure causes dendritic spine loss in female mice’s PFC

## Graphical abstract

**Figure:**
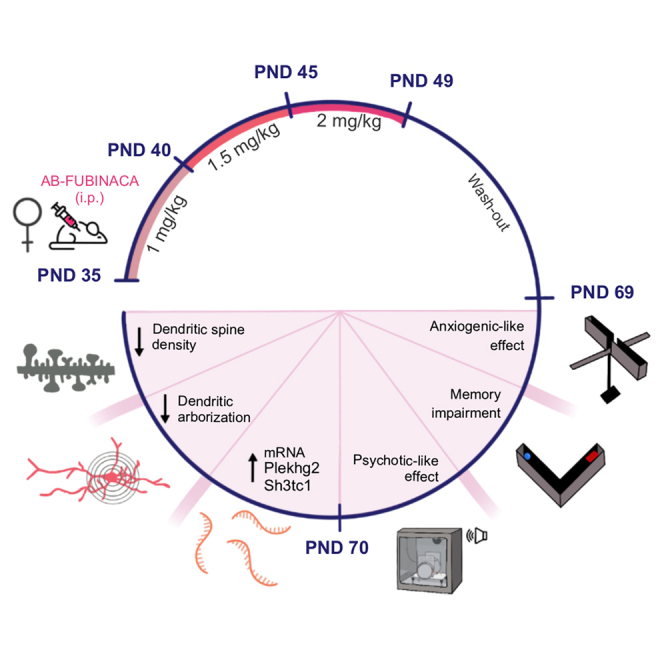

## Introduction

Synthetic cannabinoids (SCBs) are emerging drugs of abuse detected in herbal incense products and sold under names such as K2 (in North America) or Spice (in Europe), primarily through the internet.^1^ Compared to Δ^9^-tetrahydrocannabinol (Δ^9^-THC), the principal psychoactive ingredient of cannabis, SCBs are potent full agonists of brain cannabinoid receptors, leading to more potent effects than those produced by Δ^9^-THC.^2^ Indeed, the consumption of SCBs has been increasingly associated with severe intoxications and even deaths, thus representing a global public health problem.^3^ Adolescents and young adults show the highest rate of SCB use^4^ which is of particular concern because adolescence is a critical period in brain development and maturation.^5^ Despite the dramatic increase of SCB use among young adults^6^^,^^7^ their impact on brain function and behavior in adulthood represents an important unanswered health question.

SCBs can be categorized into distinct groups and subgroups depending on their chemical structure. In 2010, naphthoylindoles, such as JWH-018 and JWH-073, and cyclohexylphenols, such as CP-47,497, were the primary SCBs found in seized Spice/K2 products.^8^ Research into the mechanisms of SCBs-related toxicity has mostly focused on these naphthoylindoles (e.g., JWH series) while the toxicological profiles and impact on the behavior of other SCBs classes (e.g., indole- and indazole-based SCBs) have been hardly studied.^2^

AB-FUBINACA is a member of the indazole carboxamide family of SCBs which includes other compounds that have been linked to deaths in the United States such as AB-CHMINACA or AB-PINACA.^9^ AB-FUBINACA presents potent affinity for the CB1 cannabinoid receptor (CB1R) (Ki = 0.9 nM).^10^ In animal models, AB-FUBINACA reproduced the typical “tetrad” effects of Δ^9^-THC which are hypothermia, analgesia, hypolocomotion, and catalepsy.^11^ Regarding anxiety, the acute administration of AB-FUBINACA in adult mice induced anxiolytic- or anxiogenic-like effects depending on the dose.^12^ This SCB has also been shown to produce physical dependence^13^ and impair recognition memory^14^ after chronic administration in mice. However, lasting neurobiological consequences induced by adolescent exposure to AB-FUBINACA remain poorly understood.

In this study, we investigated the long-term consequences of adolescent exposure to AB-FUBINACA on key neurobehavioral responses associated with SCB toxicity in humans. Anxiety, fear extinction, recognition memory, sociability, depression, and sensorimotor gating were evaluated in male and female mice after treatment with AB-FUBINACA during adolescence. Given the behavioral results observed, possible structural plasticity alterations and RNA-Seq profile were analyzed in the prefrontal cortex of female mice exposed to this SCB.

## Results

### Long-term consequences in adolescent mice exposed to AB-FUBINACA on the extinction of fear and anxiety

**Figure 1:**
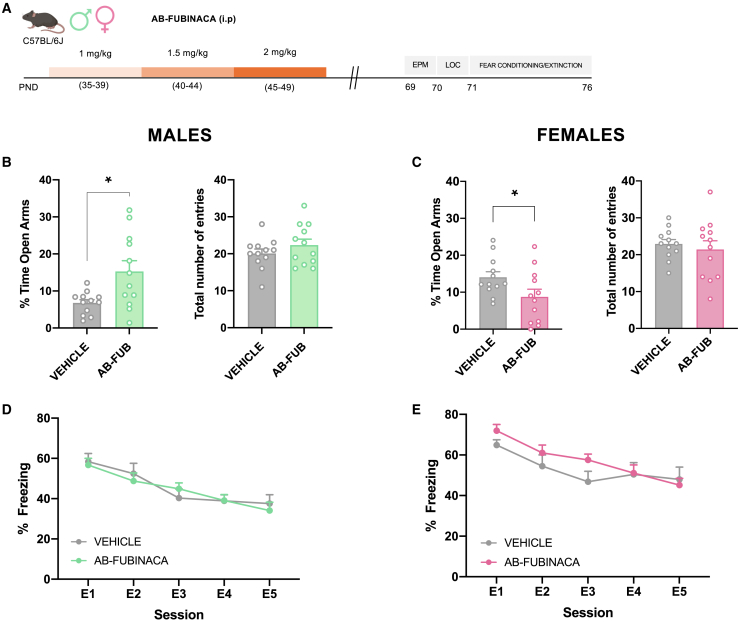
AB-FUBINACA treatment during adolescence alters anxiety-like behavior in a sex-dependent manner (A) Schematic representation of experimental design. (B–E) Effects of adolescent exposure to AB-FUBINACA (PND 35–39: 1 mg/kg, PND 40–44: 1.5 mg/kg, and PND 45–49: 2 mg/kg) or vehicle in anxiety-like behavior in the EPM (B, C) and fear extinction (D, E) in male (B, D) and female (C, E) mice (*n* = 12 mice per group). Percentage of time spent in the open arm and total number of entries are shown for the EPM. Time course of the freezing levels scored during cued fear extinction trials is shown for fear memory processing. Data are expressed as mean ± SEM. ∗*p* < 0.05 (comparison between AB-FUBINACA and vehicle; Student’s t test). PND postnatal day, EPM elevated plus maze, LOC locomotion, E1-E5 extinction trials.

Adolescent male and female mice were treated with increasing doses of AB-FUBINACA for 15 days (PND 35–39: 1 mg/kg, PND 40–44: 1.5 mg/kg, and PND 45–49: 2 mg/kg) (Figure 1A). Body weight was daily evaluated along with AB-FUBINACA treatment. The weight gain of mice treated with AB-FUBINACA was lower than those exposed to the vehicle in both sexes (Figure S1) (interaction day x treatment: F_14,1162_ = 10.79, *p* < 0.0001 and F_14,1372_ = 4.99, *p* < 0.0001, for male and female mice, respectively), in agreement with previous reports evaluating effects of adolescent cannabinoid exposure. Locomotor activity, anxiety-like behavior, and fear memory processing were analyzed 20 days after the finishing of the AB-FUBINACA treatment (Figure 1A). No changes in locomotion were observed in either males or females (Figure S2). By using the elevated plus maze (EPM), AB-FUBINACA induced an opposite effect on anxiety depending on the sex. Anxiolytic- or anxiogenic-like effects (*p* < 0.05) were observed, respectively, in male and female mice, without changes in the total number of entries (Figures 1B and 1C). Aversive memory processing was evaluated by a cued fear conditioning and extinction paradigm. The administration of AB-FUBINACA did not alter fear extinction in both males (Figure 1D) and females (Figure 1E). However, fear conditioning was higher in females (interaction cue x treatment: F_2,40_ = 4.91, *p* < 0.05) treated with AB-FUBINACA in comparison with controls, while no differences were observed in the case of male mice (Figure S3). These results suggest the existence of sex-specific effects in anxiety due to AB-FUBINACA exposure during adolescence.

### Long-term consequences in adolescent mice exposed to AB-FUBINACA on non-emotional memory, sociability, and depression

**Figure 2:**
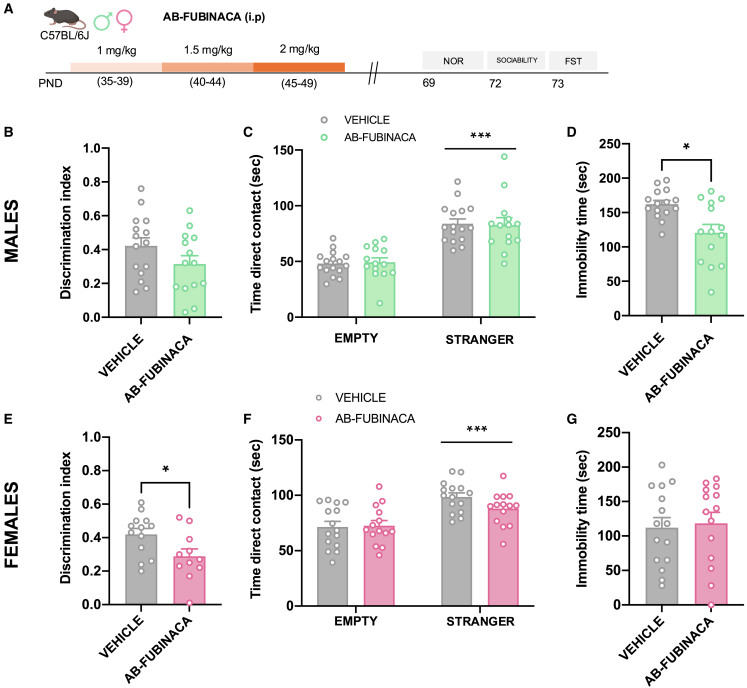
AB-FUBINACA treatment during adolescence alters novel object recognition memory and depressive-like behavior in a sex dependent manner (A) Schematic representation of experimental design. (B–G) Effects of adolescent exposure to AB-FUBINACA (PND 35–39: 1 mg/kg, PND 40–44: 1.5 mg/kg, and PND 45–49: 2 mg/kg) or vehicle in memory in the NOR (B, E), sociability in the three chamber test (C, F), and depressive-like behavior in the FST (D, G) in male (B, C, D) and female (E, F, G) mice (*n* = 11–15 mice per group). The discrimination index is shown for the NOR, total time in direct contact with each compartment is shown for sociability test, and immobility time is shown in the FST. Data are expressed as mean ± SEM. ∗*p* < 0.05, ∗∗∗*p* < 0.001 (comparison between AB-FUBINACA and vehicle group; two-way ANOVA, compartment (C, F); Student’s t test (D, E). PND postnatal day, NOR novel object recognition, FST forced swimming test.

To study possible durable alterations in non-emotional memory, sociability, and depression, mice were exposed to a similar protocol of AB-FUBINACA administration (Figure 2A). By using the novel object recognition test (NOR), memory was not affected in male mice as showed the lack of changes in the discrimination index between control and treated animals (Figure 2B). Interestingly, the discrimination index was lower in females exposed to AB-FUBINACA during the adolescent period in comparison with controls (*p* < 0.05) (Figure 2E), indicating the existence of memory deficits in these animals. Total exploration time was not altered in any of the different experimental groups (Figure S4). On the other hand, social behavior was not affected in either males or females as revealed by similar contact times between controls and mice treated with AB-FUBINACA (Figures 2C and 2F). Finally, a decrease in immobility time was observed in adult male mice treated with AB-FUBINACA during adolescence (*p* < 0.05) (Figure 2D) in the forced swimming test (FST), while this difference was not present in the case of female mice (Figure 2G). Therefore, adolescent exposure to AB-FUBINCA induced different effects on memory and depressive-like behavior in adult male and female mice.

### Long-term consequences in adolescent mice exposed to AB-FUBINACA on sensorimotor gating

**Figure 3:**
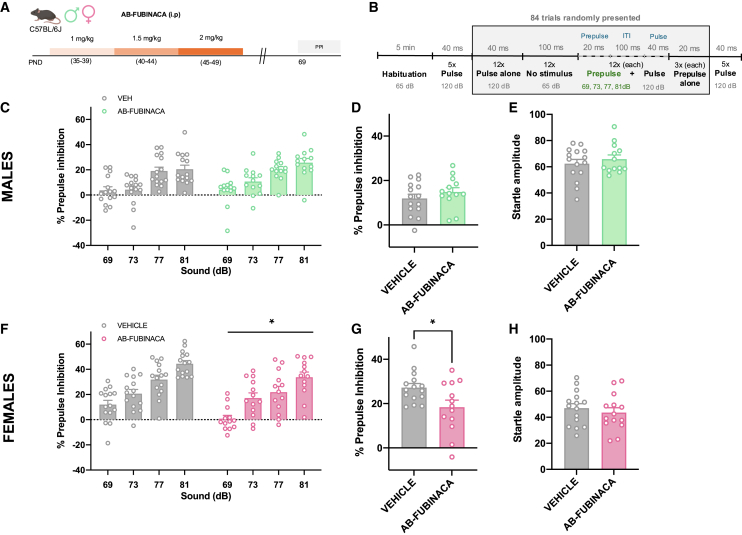
AB-FUBINACA treatment during adolescence alters sensorimotor gating in a sex-dependent manner (A) Schematic representation of experimental design. (B) Graphic diagram of the PPI protocol. (C–H) Effects of adolescent exposure to AB-FUBINACA (PND 35–39: 1 mg/kg, PND 40–44: 1.5 mg/kg, and PND 45–49: 2 mg/kg) or vehicle in sensorimotor gating in male (C, D, E) and female (F, G, H) mice (*n* = 13–15 mice per group). Percentage of prepulse inhibition (C, F), mean of the percentage of prepulse inhibition (D, G), and startle response amplitude (E, H) are shown. Data are expressed as mean ± SEM. ∗*p* < 0.05 (comparison between AB-FUBINACA and vehicle group; two-way ANOVA with repeated measures, treatment (F); Student’s t test (G). PND postnatal day, PPI prepulse inhibition test, dB decibels, ITI inter-trial interval.

Impairments of prepulse inhibition (PPI) of the startle reflex, a sensorimotor gating process, are observed in patients with schizophrenia and are considered a marker of psychotic-like behavior.^15^ We studied possible PPI alterations in both adult male and female mice treated with AB-FUBINACA during the adolescent period (Figure 3A). Details of the experimental protocol of PPI used are presented in Figure 3B. AB-FUBINACA exposure did not modify the PPI of the startle reflex in males (Figures 3C and 3D). The magnitude of the startle reflex was also not altered by AB-FUBINACA injection (Figure 3E) in these mice. Notably, a significant decrease in PPI of the startle reflex was revealed in female mice (treatment effect: F_1,26_ = 6.04, *p* < 0.05) (Figure 3F). An overall reduction of PPI due to AB-FUBINACA exposure was also observed when representing the mean PPI score (*p* < 0.05) (Figure 3G). This effect was independent of baseline changes in startle amplitude (Figure 3H), ruling out an impact of startle reaction in the modifications of PPI observed. To evaluate whether the deficits observed in PPI would be preserved in the long term, we chose a time point quite far from the initial measurement (80 days after ending treatment with AB-FUBINACA). However, the deficits in PPI were not maintained at this point (Figure S5). Taken together, these results suggest a sex-dependent alteration in sensorimotor gating due to the adolescent exposure to the SCB AB-FUBINACA.

**Figure 4:**
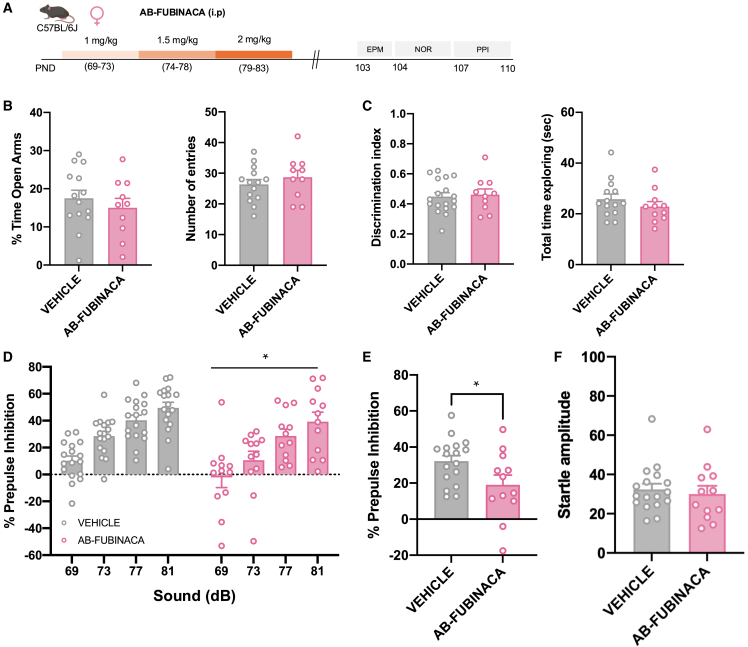
AB-FUBINACA treatment during adulthood alters sensorimotor gating, but neither anxiety-like behavior nor memory, in female mice (A) Schematic representation of experimental design. (B–F) Effects of adult exposure to AB-FUBINACA (PND 69–73: 1 mg/kg, PND 74–78: 1.5 mg/kg, and PND 79–83: 2 mg/kg) or vehicle in anxiety-like behavior in the EPM (B), memory in the NOR (C), and sensorimotor gating (D, E, F) in female mice (*n* = 10–17 mice per group). Percentage of time spent in the open arm and total number of entries are shown for the EPM, the discrimination index and total time of exploration are represented for the NOR. Percentage of prepulse inhibition (D), mean of the percentage of prepulse inhibition (E), and startle response amplitude (F) are also shown. Data are expressed as mean ± SEM. ∗*p* < 0.05 (comparison between AB-FUBINACA and vehicle group; two-way ANOVA with repeated measures, treatment (D); Student’s t test (E). PND postnatal day, EPM elevated plus maze, NOR novel object recognition, PPI prepulse inhibition test, dB decibels.

To elucidate whether an immature brain represents a period of development more susceptible to the effects of AB-FUBINACA, we evaluated the consequences of the treatment with this SCB directly in adult female animals (Figure 4A). Possible changes in anxiety, non-emotional memory, and sensorimotor gating were assessed 20 days following the last injection of AB-FUBINACA (Figure 4A), as previously studied during the adolescent exposure to this drug. Notably, no differences in the EPM (Figure 4B) and NOR (Figure 4C) tests were observed between vehicle and AB-FUBINACA groups. These results indicate that adolescence represents a sensitive window for the harmful consequences of AB-FUBINACA exposure on anxiety and memory. In contrast, AB-FUBINACA administration in adult females induced a decrease in PPI (Figures 4D and 4E), without changes in the magnitude of startle reflex (Figure 4F), as previously observed in adolescent female mice treated with this SCB. Therefore, the alterations induced by AB-FUBINACA in sensorimotor gating in females were independent of the temporal window of administration and emphasized the detrimental consequences of AB-FUBINACA exposure in the appearance of psychotic-like symptoms.

### Transcriptome analysis in adult female mice exposed to AB-FUBINACA during adolescence

**Figure 5:**
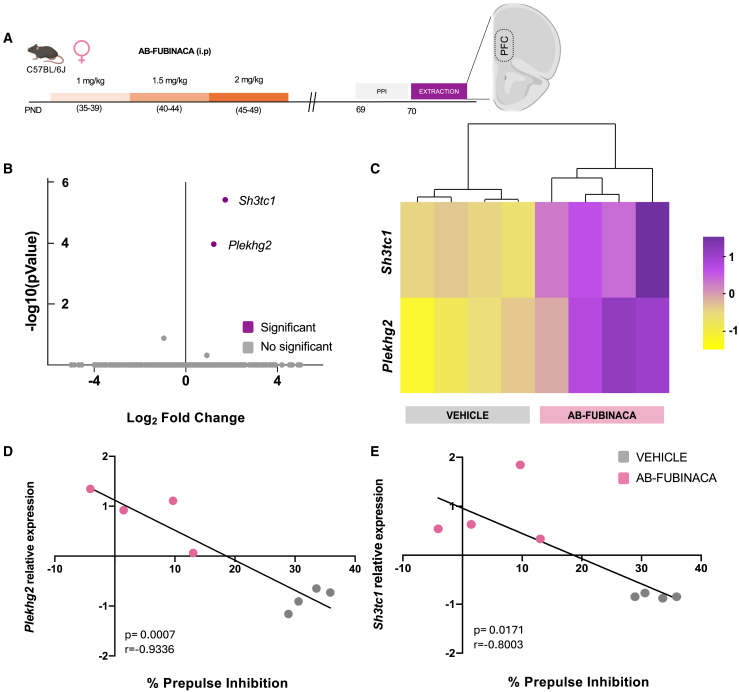
AB-FUBINACA treatment during adolescence induces an up-regulation of PLEKHG2 and SH3TC1 genes in the prefrontal cortex of adult females (A) Schematic representation of experimental design. (B) Volcano plot summarizing DEGs of AB-FUBINACA vs. vehicle treated mice (*n* = 4 mice per group). (C) DEGs in each AB-FUBINACA and vehicle treated mouse clustered with a heatmap. (D and E) Correlation between percentage of prepulse inhibition and *Plekhg2* (D) and *Sh3tc1* (E) relative expression (Pearson correlation coefficient). PND postnatal day, PPI prepulse inhibition test, PFC prefrontal cortex, DEGs differentially expressed genes.

The prefrontal cortex is a brain area directly related to the modulation of sensorimotor gating.^16^ We next used RNA-Seq to examine the molecular profile of this brain region in female mice exposed to AB-FUBINACA during adolescence (Figure 5A), given the deficits previously observed in PPI of the startle reflex in these animals. Prefrontal cortex samples used for the transcriptome analysis were obtained from the set of animals previously subjected to PPI. RNA-Seq identified two differentially expressed genes (DEGs) (adjusted *p* < 0.05 and cutoff of 2-fold change) which were upregulated in mice injected with AB-FUBINACA (Figure 5B). These two DEGs (*Plekhg2* and *Sh3tc1*) in each vehicle- and AB-FUBINACA-treated mouse were clustered with a heatmap (Figure 5C). Principal component analysis (PCA) is shown in Figure S6. Significant negative correlations between the percentage of PPI and the relative expression of *Plekhg2* (*p* < 0.001) (Figure 5D) and *Sh3tc1* (*p* < 0.05) (Figure 5E) were found in the prefrontal cortex of female mice treated with vehicle or AB-FUBINACA.

### Structural plasticity analysis in adult female mice exposed to AB-FUBINACA during adolescence

**Figure 6:**
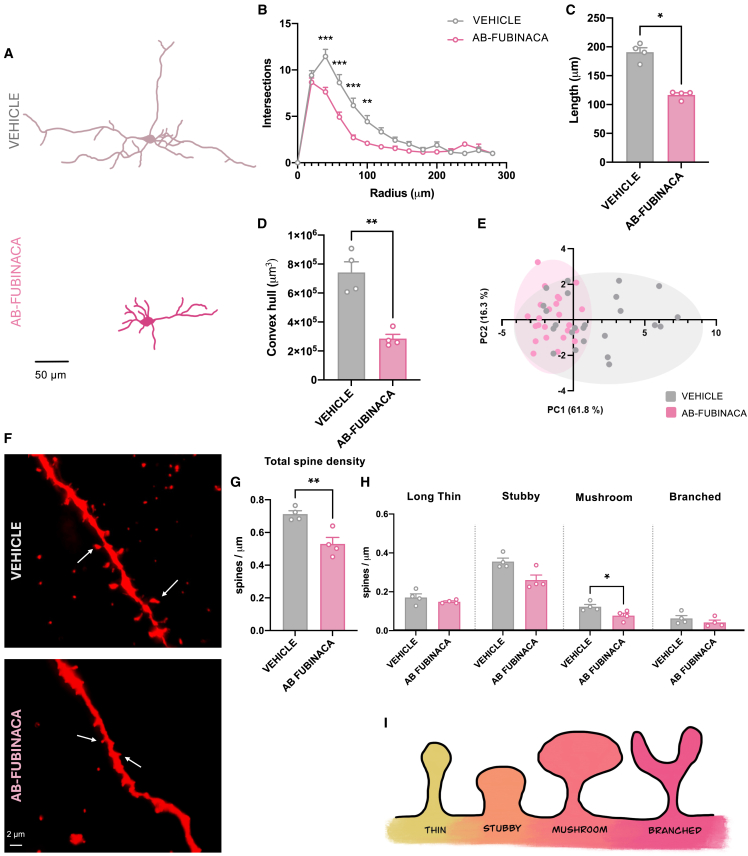
AB-FUBINACA treatment during adolescence induces alterations in dendritic arborization and dendritic spine density in the prefrontal cortex of adult females Effects of treatment with AB-FUBINCA or vehicle during adolescence in dendritic arborization (A-E) and dendritic spine density (F-I) in the prefrontal cortex of adult female mice. (A) Representative traces of reconstructed neurons in AB-FUBINACA or vehicle-treated female mice. Scale bar = 50 μm. (B) Sholl analysis represented by the number of intersections every 20 μm. (C) Total dendritic length. (D) Volumetric data of neuron dendrites in μm3. (E) Plot comparing the total number of reconstructed neurons (*n* = 6 neurons/mouse and *n* = 4 mice per group) across principal components PC1 and PC2. (F) Representative images of apical dendritic spines from AB-FUBINACA and vehicle treated female mice. Scale bar = 2 μm. (G) Total spine density of apical dendrites in AB-FUBINACA and vehicle treated female mice (n = 4–5 neurons/mouse and *n* = 4 mice per group). (H) Spine density grouped according to their morphological characteristics in apical dendrites. (I) Schematic representation of the morphological classification of the dendritic spines. Data are expressed as mean ± SEM. ∗*p* < 0.05, ∗∗*p* < 0.01 and ∗∗∗*p* < 0.001 (comparison between AB-FUBINACA and vehicle group; mixed-model ANOVA, interaction treatment x radius (B); Mann-Whitney U test (C); Student’s t test (D, G, H). PC principal components 1 and 2.

*The plekhg2* gene, which encodes a Rho family-specific guanine nucleotide exchange factor, has been recently associated with dendritic spine morphology formation and corticogenesis.^17^ Moreover, Rho family guanosine triphosphatases (GTPases), including Cdc42, are known to regulate various cellular processes, such as morphology, gene transcription, proliferation, and migration through actin cytoskeletal rearrangement. Adolescent AB-FUBINACA exposure did not modify Cdc42 activity in the prefrontal cortex of female adults (Figure S7), suggesting that other Rho GTPases could be regulated by *Plekhg2*. We next analyzed the existence of possible structural plasticity alterations in pyramidal neurons of the prefrontal cortex of adult female mice treated with AB-FUBINACA during adolescence. Sholl analysis showed lower dendritic arborization in AB-FUBINACA-treated mice compared to controls as revealed by mixed-model ANOVA (treatment effect: F_1,46_ = 6.01, *p* < 0.05; interaction treatment x radius: F_14,370_ = 4.68, *p* < 0.0001) (Figures 6A and 6B). Total length dendrites were shorter in mice exposed to the SCB than in controls (*p* < 0.05) (Figures 6A, 6C, and S8A). This effect was revealed as the branching order increased (Figures S8B–S8D). Moreover, convex hull volume was also lower (*p* < 0.01) in female mice treated with AB-FUBINACA (Figure 6D). PCA identified different groupings of the neuronal populations, by using an unbiased approach (Figure 6E). Total apical (Figures 6F and 6G), but not basal (Figures S8E and S8F), dendritic spine density decreased in female mice exposed to AB-FUBINACA (*p* < 0.01). Moreover, the density of mushroom (mature) spines (*p* < 0.05) was lower in mice treated with the synthetic cannabinoid in comparison with controls (Figure 6H). No changes were observed in long thin, stubby, and branched spines (Figure 6H). As a whole, these results indicate that adolescent AB-FUBINACA exposure in female mice involves changes in dendritic arborization and in the density of mature spines in the prefrontal cortex of adult animals. These alterations were associated with the presence of psychotic-like symptoms, as revealed by the deficits in the PPI test.

## Discussion

Our results show important sex-dependent long-term behavioral effects due to adolescent exposure to the SCB AB-FUBINACA, present in Spice/K2 preparations. Notably, treatment with AB-FUBINCA during adolescence induced impairments of PPI of the startle reflex in adult female mice, but not in males. These deficits were also manifested when the cannabinoid was directly administered in adult female animals, suggesting a potent effect of this compound to produce psychotic-like symptoms. The presence of PPI alterations was associated with a decrease in the density of mature dendritic spines and a lower dendritic arborization in the prefrontal cortex of cannabinoid-exposed mice.

The weight gain of mice treated with AB-FUBINACA was lower than controls, as previously reported in the case of other cannabinoids such as Δ^9^-THC^18^^,^^19^ or JWH-018.^20^ An anxiogenic-like effect induced by AB-FUBINACA could explain the changes in body weight. Indeed, one week after the finishing of the treatment, a higher level of anxiety-like behavior in adolescent rodents exposed to Δ^9^-THC compared to controls was found in the EPM test in previous studies.^18^ Nonspecific inhibition of ingestion, secondary to the potential sedative effects of AB-FUBINACA could also be involved in this effect on body weight.

The administration of AB-FUBINACA during adolescence affected anxiety in a sex-dependent manner. Thus, anxiolytic-like effects were observed in adult males, while anxiogenic-like responses were revealed in female adult mice. Consistent with this, fear conditioning was higher in females treated with AB-FUBINACA compared to controls, although no differences in fear memory acquisition were observed in adult female mice exposed to Δ^9^-THC during adolescence in a previous study.^21^ Controversial data have been shown regarding the effects of adolescent cannabinoid exposure on anxiety. In general, by using the EPM, most of the studies have not shown differences in anxiety in adult male^21^^,^^22^^,^^23^ or female^22^^,^^23^ rodents due to adolescent Δ^9^-THC exposure, although an anxiogenic-like effect was observed in adult male mice in another study.^24^ Regarding the effects of SCBs, male adolescent rats treated with AB-FUBINACA did not show changes in anxiety when reaching adulthood by using the emergence test.^25^ However, an anxiogenic trend was observed in adult female, but not male, animals exposed to the SCB JWH-018 during adolescence.^20^ On the other hand, anxiety disorders characterized by pathological fear, such as post-traumatic stress disorder and phobias, are associated with extinction deficits of aversive memories. Under our experimental conditions, cued fear extinction was not affected in either male or female mice by adolescent exposure to AB-FUBINACA. In agreement, the administration of Δ^9^-THC or JWH-018 during adolescence did not alter fear extinction in male and female adult mice.^20^^,^^21^

The detrimental effects of adolescent exposure to synthetic or natural cannabinoids on novel object recognition are well-known.^26^ Interestingly, similar treatments during adulthood did not produce such long-term deleterious effects.^27^^,^^28^ Accordingly, we observed a memory impairment in adult female, but not male, mice treated with AB-FUBINACA during adolescence, while this effect was not preserved when a similar experimental procedure was carried out in adult animals. However, a recent study has shown an impairment of the recognition memory in adult mice exposed to chronic AB-FUBINACA administration,^14^ although this effect was observed in the short-term, 24 h after the last drug injection. On the other hand, an antidepressant-like effect was found in male mice exposed to AB-FUBINACA during adolescence, as revealed by the decrease in the immobility time in the FST test. Notably, both preclinical and epidemiological data suggest that females are more vulnerable than males to the deleterious effects of adolescent cannabinoid exposure on mood,^29^ although no alteration in depressive-like behavior was observed in female mice in our study.

PPI of the startle reflex is a classic preclinical model of sensorimotor gating assessment which involves a sensory filtering mechanism to prevent sensory information overload.^30^ Deficits in PPI have been observed in several psychiatric disorders, particularly in schizophrenia.^31^ Adolescent AB-FUBINACA exposure induced a reduction of PPI of the startle reflex in female, but not male, adult mice. Notably, this detrimental effect was conserved when the same drug administration regimen was performed directly in adulthood, suggesting that this SCB has an important potential to produce psychotic-like symptoms. Different results have been described regarding the effects of cannabinoid exposure during adolescence on PPI in rodents. Thus, Δ^9^-THC or JWH-018 administration induced persistent PPI deficits in adult male rats^32^^,^^33^ and mice,^20^ respectively. However, an increase in PPI has been also observed in male mice treated with Δ^9^-THC during adolescence.^34^ On the other hand, the chronic administration of JWH-018 in adult rodents did not induce changes in PPI in male rats^35^ while causing sensorimotor gating deficits in male mice.^36^ Interestingly, we found that the behavioral alterations induced by adolescent exposure to AB-FUBINACA were modulated by sex, being females more vulnerable than males. Thus, anxiogenic-like responses, memory impairment, and PPI deficits were observed in females, but not in male mice. Despite the well-accepted observation that several neuropsychiatric disorders are sex-related, few articles have dealt with sex vulnerability to adolescent cannabinoid exposure, both at the preclinical and clinical levels. In this sense, animal models seem to suggest that females are more sensitive to the effects of cannabinoids than males in the emotional sphere.^37^ Sex has been described as a major factor modulating the pharmacokinetic and brain activity effects of Δ^9^-THC in adolescent rats.^38^ Future experiments will be required to study the influences of these and other factors (i.e.,:., tolerance from repeated injections, hormonal status), in the sex-dependent effects induced by AB-FUBINACA.

The prefrontal cortex is a brain area directly related to the modulation of sensorimotor gating.^16^ By using RNA-Seq, we found that *Sh3tc1* and *Plekhg2* genes were up-regulated in the prefrontal cortex of female adults exposed to AB-FUBINACA during adolescence. *The plekhg2* gene encodes a guanine nucleotide exchange factor promoting GDP/GTP exchange to activate Rho GTPases, including Rac and Cdc42.^39^ Abnormalities of the *Plekhg2* gene are involved in postnatal microcephaly and intellectual disability.^40^ Specifically, all these patients suffered with profound mental retardation, dystonia, postnatal microcephaly, and a suggestive neuroimaging pattern which consisted of paucity of white matter and dorsal tegmental tracts, and particularly pons, involvement.^40^ Interestingly, the *Plekhg2* gene has been recently shown to be essential for axon, dendrite, and synapse development in mouse cortical neurons *in vivo*.^17^ The physiological and balanced expression of this gene, but not a defect or an excess of this expression, probably will be important for a correct biological function. Congruent with this, we observed a lower dendritic arborization and a reduction of total length dendrites in prefrontal cortex pyramidal neurons of adult female mice exposed to AB-FUBINACA during the adolescent period. Furthermore, a decrease in the density of total and mushroom-shaped (mature) dendritic spines was also revealed in the same brain region in this group of animals. These observations provide novel evidence of a potential link between prefrontal cortex dysmorphology and PPI alterations, which in turn could contribute to the presence of psychotic-like symptoms due to AB-FUBINACA adolescent exposure. In this sense, adolescent exposure to the SCB CP55,940 in male rats leads to reduced basal dendrite arborization in pyramidal neurons in the prefrontal cortex of adults,^31^ suggesting that cannabinoids may impede the structural maturation of neuronal circuits in this brain region, thus inducing impaired cognitive function in adulthood. Reduced dendritic spine density in the hippocampal dentate gyrus was also associated with the impairment of spatial working memory in adult male rats treated with Δ^9^-THC during adolescence.^41^

In summary, our findings reveal long-term behavioral alterations associated with chronic adolescent exposure to AB-FUBINACA, a member of the indazole carboxamide family of SCBs. Understanding the detrimental consequences of SCB abuse in the young population is crucial to developing drug-specific treatments for intoxication and effective education and prevention programs.

### Limitations of the study

We show important long-term sex-dependent behavioral effects induced by adolescent exposure to AB-FUBINACA. SCBs present in Spice/K2 preparations seem to produce more detrimental effects than those produced by Δ^9^-THC, although no direct comparison between AB-FUBINACA and Δ^9^-THC was carried out in this study.

We used similar doses of AB-FUBINACA in male and female mice. Testing different doses in male and female animals would be of interest to evaluate whether the behavioral differences between sexes observed are qualitative or could be affected by a particular dose range.

We identify AB-FUBINACA as a drug of abuse with a high potential to produce psychotic-like symptoms. AB-FUBINCA is frequently detected in Spice/K2 preparations, but these herbal smoking mixtures can contain diverse bioactive compounds contributing to the detrimental effects associated with their consumption in humans.

We show PPI alterations in adult female mice, but not in males, exposed to AB-FUBINCA during adolescence. Given these results, we studied potential changes in structural plasticity in the prefrontal cortex of female mice. Additional studies are necessary to evaluate possible modifications in dendrite morphology in the prefrontal cortex of male mice treated with this SCB.

## Resource availability

### Lead contact

Further information should be directed to and will be fulfilled by the lead contact, Fernando Berrendero (fernando.berrendero@ufv.es).

### Materials availability

This study did not generate unique reagents.

### Data and code availability

• The RNA-seq data have been deposited at the NCBI SRA database (ID number: PRJNA 1167322) and are publicly available as of the date of publication. Accession number is listed in the key resources table.
• This article does not report the original code.
• Any additional information required to reanalyze the data reported in this article is available from the lead contact upon request.

## Acknowledgments

This work was supported by “10.13039/100016658Plan Nacional sobre Drogas” [#2019I024], MICIU/AEI/10.13039/501100011033/ Grant [PID2020-116579RB-I00], MICIU/AEI/10.13039/501100011033 and FEDER, UE/ Grant [PID2023-151223OB-I00], and “Proyectos de Investigación Universidad Francisco de Vitoria (2021–2022).” CI-L is supported by a predoctoral fellowship from 10.13039/100012986Universidad Francisco de Vitoria. M.P-R is supported by a predoctoral fellowship from MICIU. We would like to thank the confocal microscopy facility at Centro de Biología Molecular Severo Ochoa (CBMSO) for technical assistance, and Dra Esther Martín for her help with biochemical experiments.

## Author contributions

CI-L, MP-R, MT-B, RM-T, and IP-P performed behavioral and biochemical experiments. MA-A analyzed images. FB and IP-P conceived the idea. FB funded this project. CI-L and FB wrote the article. All co-authors edited and approved the final version of the article.

## Declaration of interests

The authors declare no competing interests.

## STAR★Methods

### Key resources table

**Table:** 

REAGENT or RESOURCE	SOURCE	IDENTIFIER
**Chemicals, peptides, and recombinant proteins**		

AB-FUBINACA	Cayman	CAY-14039; CAS: 1185282-01-2

**Critical commercial assays**		

RiboPureTM Kit	Invitrogen	Cat#10107824
G-LISA Activation Assay	Cytoskeleton Inc.	Cat#BK127
FD Rapid GolgiStain kit	FD NeuroTechnologies, Inc	Cat#PK401A

**Deposited data**		

RNAseq	NCBI SRA	PRJNA 1167322

**Experimental models: Organisms/strains**		

Male and female C57BL/6J mice	Charles Rivers	632C57BL/6J

**Software and algorithms**		

GraphPad Prism 9.2.0	Graphpad	https://www.graphpad.com/
STATISTICA ®	StatSoft	https://www.statsoft.de
Sholl Analysis – Neuroanatomy plugin – FIJI	FIJI free software	https://fiji.sc
Dendritic spine– Filament tracer tool - IMARIS	Oxford Instruments	BPA-IM-Tracer97
PCA – FactoMineR – R package version 2.11	r- project free software	https://doi.org/10.32614/CRAN.package.FactMineR

**Other**		

Chamber for fear conditioning and PPI test	PanLab	Cat#LE116
Actimetry	Cibertec	Not specified
Confocal microscope	Zeiss, CLSM	LSM 900

### Experimental model and study participant details

#### Animals

Adolescent male and female, and adult female C57BL/6J mice (Charles River, France) were used in these experiments. Animals were housed 4-5 per cage in a temperature (21 ± 1°C)-and humidity (55 ± 10%)-controlled room under a 12 h light/dark cycle. Food and water were available *ad libitum*. All behavioral studies were performed during light period. Experimental procedures were conducted in accordance with the guidelines of the European Communities Directive 2010/63/EU and Spanish Regulations RD 1201/2005 and 53/2013 regulating animal research and approved by the local ethical committee (CEEA-UFV) (283.2/21).

### Method details

#### Drugs

AB-FUBINACA (Cayman Chemical) was prepared in a 5% ethanol, 5% Tween-80 and 90% saline solution and was intraperitoneally (i.p.) administered at doses of 1, 1.5 and 2 mg/kg (10 ml/kg of body weight). Dosage was based on previous studies.^10^^,^^13^^,^^14^

#### Experimental designs

##### AB-FUBINACA treatment in adolescent mice

We evaluated long-term effects due to adolescent exposure to AB-FUBINACA on anxiety-like behavior, cued fear conditioning and extinction, object memory, sociability, depressive-like behavior and prepulse inhibition (PPI) of the startle reflex in both male and female mice. The temporal boundaries of adolescence, a vulnerable period to the central effects of drugs^5^^,^^26^ are not precisely defined in either humans or rodents.^26^ Thus, based on previous studies,^20^^,^^21^ the treatment started at PND 35. Mice were i.p. treated with increasing doses of AB-FUBINACA (PND 35-39: 1 mg/kg, PND 40-44: 1.5 mg/kg, and PND 45-49: 2 mg/kg) or vehicle for 15 days. Long-term effects were analyzed 20 (PND 69) days after the end of the treatment. The interval of time between adolescent treatment and the different behavioral assays were based on previous reports.^20^^,^^21^ Behavioral studies were carried out in 6 different batches (3 per sex) as described in Figures 1A, 2A, and 3A. The first was used for locomotion, anxiety and fear extinction experiments (males, n = 12; females, n =12), the second, for performing object recognition, sociability and forced swimming tests (males, n = 14-15; females, n = 11-13) and the third for the experiments of PPI (males, n = 13-15; females, n = 13–15). Tissues were obtained 24 h after the PPI test to carry out biochemical experiments. For RNAseq experiments, the number of mice used was 4 per group. An additional experiment was performed for Golgi staining (n = 4 mice per group). The number of animals used in this study is in the usual range of similar experiments previously published.^42^^,^^43^

##### AB-FUBINACA treatment in adult mice

To determine if adolescence is a period of vulnerability to the effects observed in AB-FUBINACA treated mice, a similar protocol was conducted in adult mice (Figure 4A).

Aligning with the prior experimental framework where PND 69 marked adulthood, intraperitoneal injections of AB-FUBINACA or vehicle started at this point, increasing the doses as previously described (PND 69-73: 1 mg/kg, PND 74-78: 1.5 mg/kg, and PND 79-83: 2 mg/kg). Behavioral evaluation started 20 days after the end of the treatment (PND 103), as described in Figure 4A.

#### Behavioral experiments

##### Elevated plus maze

Anxiety-like behavior was assessed by using an elevated plus maze (EPM), which consisted in four arms (25 × 5 cm) set in cross from a central square (5 × 5 cm) and raised 30 cm from the ground. Two opposite arms were delimited by vertical walls (closed arms), although the two other arms had unprotected edges (open arms). The apparatus was indirectly illuminated with 40-50 lux. The 5 min test was recorded through a videocamera located on top of the maze. Results are expressed as total entries to the open and closed arms, and the percentage of time spent in the open arms with respect to the total amount of time spent in both closed and open arms.

##### Cued fear conditioning and extinction

Training and testing were performed as in preceding experiments with slight modifications.^20^^,^^21^ Mice were individually placed in the chamber (LE116, Panlab, Harvard Instruments) made of black walls with a transparent front door. The box (25 × 25 × 25 cm) was located inside a soundproof module to provide background noise and to reduce outside sound. The chamber floor was formed by parallel metal bars (2 mm of diameter and 6 mm spaced) connected to a shock generator (LE100- 26 module, Panlab, Harvard Instruments). A high-sensitivity weight transducer (load cell unit) was used to record the signal generated by the animal movement intensity. The software PACKWIN V2.0 automatically quantified the percentage of immobility for each experimental phase. Between animal trials, the chamber was cleaned with 70% ethanol and water to avoid olfactory cues. The conditioning session consisted of a 180 sec habituation followed by three cue tones (3 Hz, 80 dB) of 30 sec long. Each cue (conditioned stimulus, CS) co-terminates with a 0.7 mA foot-shock of 1 sec duration (unconditioned stimulus, US). The interval between cues lasted 10 sec. Fear extinction sessions (E1-E5) were performed 24, 48, 72, 96 and 120 h after the conditioning day in a novel environment (white walls, transparent cylinder, and smooth floor). During E1, mice were habituated to the new context for 180 sec, whereas in E2-E5 this acclimatation period was reduced to 60 sec. Then, mice were re-exposed to the CS (4 cue tones, 30 sec long, 10 sec between tones). Fear memory was assessed as the mean percentage of time that mice spent freezing during the 4 cue tones of each extinction session. Freezing behavior, a rodent’s natural response to fear, was automatically recorded and defined as complete lack of movement, except for breathing for more than 800 ms. Data from fear extinction were expressed as percentage of freezing behavior.

##### Locomotion

Changes in locomotor activity were assessed by using activity boxes (27 × 27 × 21 cm, Cibertec). Mice were individually placed in locomotor cages with low luminosity. Activity was measured as the total number of times the animal crossed an infrared beam during 15 min.

##### Novel object recognition test

Object-recognition memory was performed by using a V-shaped maze made of matte black methacrylate with two corridors (30 × 4.5 cm and 15 cm high) joined at a 90° angle. Mice were first habituated for 9 min to the maze. The day after, animals were trained and exposed to two identical objects located at both limits of the maze and were allowed to explore for 9 min. On the test day, 24 h later, mice were again placed in the maze for 9 min, but one of the familiar objects was replaced with a novel one. Object exploration was defined as the orientation of the nose to the object at less than 2 cm. The total time the animal spent exploring each object was computed and the discrimination index was calculated as the difference between the time spent exploring novel vs familiar object divided by the total time exploring the two objects.

##### Three-chamber social interaction test

Sociability testing occurred in a three-chamber maze made of transparent methacrylate with three exact compartments (20 × 20 × 40 cm) separated by sliding doors (5 × 8 cm). After a 5 min habituation in the central chamber, the session to evaluate social affiliation/motivation started. A same-sex conspecific stranger was placed in a cylindrical cage that allows interaction in one of the side compartments, while the other compartment remained empty. The doors opened, and the mouse was allowed to explore the different compartments freely for 10 minutes. Typically, mice exhibit a preference for spending more time with other mice than alone, demonstrating sociability. Interaction times, measured as the time that the animal head was inside of a zone surrounding cylindrical cages enclosures at less than 5 cm distance, were recorded.

##### Forced swimming test

To evaluate depression-like behaviors, animals were placed in a transparent methacrylate cylinder (20 cm of diameter) filled with water (22-24°C) up to 15 cm to prevent mice from touching the bottom. They were allowed to swim freely for 6 min under normal light conditions. The cumulative duration of immobility during the last 4 min was calculated. Immobility was defined as the absence of movements except for those slights to maintain balance in the water.

##### Prepulse inhibition of startle reflex

PPI of startle reflex, a measure of sensorimotor gating, was conducted in two automated StartFear combined chambers (LE116, Panlab, Harvard Instruments) which were calibrated to ensure equivalent sensitivity and sound. Mice were daily habituated to a non-restrictive Plexiglas cylinder anchored to a high sensitivity transducer for 5 min with background white noise (65 dB) 4 days prior to test. The test started with an acclimatation period of 5 min followed by 5 pulse trials (120 dB, 40 ms) to establish baseline acoustic startle response. The experimental protocol consisted of 10 blocks with 3 or 12 trials each, randomly presented with an inter-trial interval of 10-30 s: no stimulus (12×) (65dB), pulse alone (12×) (120 dB, 40 ms), pulse precede by 4 prepulse intensities (12× each) (4, 8, 12 and 16 dB above background noise, 20 ms duration, 100 ms before pulse) and prepulse alone (3× each) (Figure 3B). Finally, 5 pulse trials were delivered. The first and last five trial pulses were excluded from the final analysis. Startle amplitude was automatically detected by PACKWIN V2.0 software. PPI was calculated as: 100 × (mean startle response – mean prepulse inhibited startle response) / (mean startle response).

#### RNA sequencing

Total RNA was purified from prefrontal cortex tissues of vehicle (n = 4) and AB-FUBINACA treated female (n = 4) mice 24 h after PPI test, with the RiboPureTM Kit (Invitrogen). RNA integrity > 7 was confirmed by TapeStation (Aligent). Sequencing libraries were prepared using TruSeq Stranded mRNA Sample Prep Kit (Illumina) following manufacturer’s instructions. Libraries were validated by using KAPA Library Quantification Kit for Illumina according to the qPCR Quantification Protocol Guide (KAPA Biosystems) and quantified by TapeStation (Aligent). Libraries were submitted to an Illumina NovaSeq and sequencing was performed using a 2 × 150 bp paired end configuration. Pseudo-alignment and quantification were then made with Salmon algorithm (reference genome GRCh38) (Patro et al.^44^). Correlation analysis, principal component study and differential expression analysis were performed with DESeq2 package (Love et al.^45^). Differential expression gene (DEG) analyses were done using the parametric Wald test, with Benjamini-Hochberg adjustment method (padj). Genes with padj < 0.05 and a cutoff of 2-fold change were considered significantly DEGs. Raw data corresponding to RNA sequencing analyses were deposited at the NCBI SRA, ID number: PRJNA 1167322.

#### G-LISA Cdc42 Activation Assay

Activity of CDC42 GTPases in prefrontal cortex tissue extract was measured by G-LISA Activation Assay (Cytoskeleton Inc.; BK127) according to the manufacturers protocol. Tissues were lysed with an appropriate lysis buffer and centrifugated (10,000 × g, 1min, 4°C). Supernatants were immediately frozen and kept at −20°C till the G-LISA Activity Assay. Protein concentration was measured by Precision Red™ Advanced Protein Assay (Cytoskeleton Inc.). Most articles that use the G-LISA kit work with cells instead of tissue, so a fine-tuning had to be carried. An amount of 3 mg/ml of sample was needed in these case, data that differs from the original protocol. The GTP-bound Cdc42 levels were performed according to the manufacturer’s protocol (Cytoskeleton Inc.) and measured with a spectrophotometer at 490 nm.

#### Golgi staining procedure

Twenty days after adolescent exposure to AB-FUBINACA (n = 4) or vehicle (n = 4), female mice were sacrificed and the whole brain was quickly and carefully removed from the skull. The Golgi staining procedure was conducted in accordance with manufacturer’s instructions, FD Rapid GolgiStain kit (FD NeuroTechnologies, Inc.; PK401A Cell Systems Biology). In summary, brains were immersed in solution A/B for 10 days in dark (with a change of the solution A/B after the first 24 h). Subsequently, they were transferred to solution C for 4–5 days prior to being sliced (with a change of the solution C after the first 24 h). Coronal sections of 160 μm thickness, spanning from 1.98 to 1.54 mm with respect to bregma for the prefrontal cortex, were obtained by using a cryostat following the protocol described by Zhong et al.^46^ After the sections dried completely on gelatin-coated slides, they were incubated in staining solution D/E for 10 min. Subsequently, stained sections were rinsed with distilled water and underwent for dehydration by a series of consecutive immersion in ethanol solutions with increasing concentrations (50, 75, 95 and 100%). Following this, samples were subjected to clearing using xylene and then mounted.

##### Image acquisition and analysis for sholl analysis

Stained sections were photographed at a 10× dry objective using a Zeiss LSM 900 confocal microscope (Zeiss, CLSM, Germany). A Z projection was made to ensure capturing the entire neuron (1 μm/stack, 16-bit, 1024 × 1024). For sholl analysis, only neurons of layer II/III of the prefrontal cortex completely impregnated within Golgi stain and that could be traced along its entire length were selected. Six independent neurons from each animal were randomly selected. To assess neuron remodeling and analysis, we used the Neuroanatomy plugin (Simple Neurite Tracer, semi-automatic tool) in FIJI (FIJI is just Image J). Finally, a principal component analysis (PCA) was performed to identify possible different groupings of the neuronal populations, by using an unbiased approach. For that, we identified 13 markers of neuron complexity: number of intersections (every 20 μm), total length (μm), number of terminal ends, total bifurcations, convex hull volume (μm^3^), average branch order, number of late-order branches, number of first, second, and third-order branches, average length of first and second-order branches, and the ratio total length –late order branches. PCA was performed using R package version 2.11 of FactoMineR.

##### Image acquisition and analysis for the morphology of dendritic spines

Section images were captured under a Zeiss LSM 900 confocal microscope, using a 60×/2× oil objective (Zeiss, CLSM, Germany) with 1.4 NA. Images were acquired through a z-plane (0.3μm/stack, 16-bit, 2048 × 2048). Secondary and tertiary dendrites of individual pyramidal neurons from layers II/III of the prefrontal cortex were selected. In addition, we chose 4-5 apical and 4-5 basal dendrites per animal for the analysis. To calculate spine density, a minimum dendrite length of 20 μm long was required. Reconstruction of dendrites and spine classification was performed by using the “FilamentTracer” tool of IMARIS software (Bitplane). Projections from dendrites were classified into 4 types based on their morphological characteristics: “stubby” were less than 0.7 μm in length, lacked a large spine head, and did not appear to have a neck; “thin spines,” larger than 0.7 μm and had elongated spine necks with small heads; “mushroom-like” were also more than 0.7 μm of length, but were characterized by a short neck and large spine head; and “branched” spines that had elongated spine necks with 2 or more spine heads.

### Quantification and statistical analysis

Normality and homoscedasticity were evaluated before the final analysis (Kolmogorov-Smirnov test and Bartlett’s test, respectively). Statistical analysis was carried out using unpaired Student t-test (with Welch’s correction when heteroscedasticity), two-way ANOVA, and two-way ANOVA of repeated measures followed by Bonferroni post hoc comparisons after significant interactions between factors. In case of missing values, a mixed-model ANOVA was performed. Nonparametric Mann-Whitney test was used when data did not fit a normal distribution. To study correlations between two variables, the Pearson's coefficient was employed. Outliers were excluded if they were >2 standard deviations from the mean. All data are expressed as mean ± SEM. A p value <0.05 was used to determine statistical significance. The statistical analyses performed for each behavioral and molecular experiment are described in Table S1. The statistical analysis was performed using STATISTICA (StatSoft) software and GraphPad Prism 9.

## References

[1] Spaderna M., Addy P.H., D’Souza D.C. (2013). Spicing things up: Synthetic cannabinoids. Psychopharmacology (Berl).

[2] Roque-Bravo R., Silva R.S., Malheiro R.F., Carmo H., Carvalho F., Da Silva D.D., Silva J.P. (2023). Synthetic Cannabinoids: A Pharmacological and Toxicological Overview. Annu. Rev. Pharmacol. Toxicol..

[3] de Oliveira M.C., Vides M.C., Lassi D.L.S., Torales J., Ventriglio A., Bombana H.S., Leyton V., Périco C.d.A.M., Negrão A.B., Malbergier A., Castaldelli-Maia J.M. (2023). Toxicity of Synthetic Cannabinoids in K2/Spice: A Systematic Review. Brain Sci..

[4] Mathews E.M., Jeffries E., Hsieh C., Jones G., Buckner J.D. (2019). Synthetic cannabinoid use among college students. Addict. Behav..

[5] Schneider M. (2013). Adolescence as a vulnerable period to alter rodent behavior. Cell Tissue Res..

[6] Miliano C., Margiani G., Fattore L., De Luca M.A. (2018). Sales and advertising channels of new psychoactive substances (NPS): Internet, social networks, and smartphone apps. Brain Sci..

[7] European Monitoring Centre for Drugs and Drug Addiction (2019). European Drug Report 2019: Trends and Developments. Publications Office. https://data.europa.eu/doi/10.2810/191370.

[8] Ford B.M., Tai S., Fantegrossi W.E., Prather P.L. (2017). Synthetic Pot: Not Your Grandfather’s Marijuana. Trends Pharmacol. Sci..

[9] Trecki J., Gerona R.R., Schwartz M.D. (2015). Synthetic Cannabinoid–Related Illnesses and Deaths. N. Engl. J. Med..

[10] Banister S.D., Moir M., Stuart J., Kevin R.C., Wood K.E., Longworth M., Wilkinson S.M., Beinat C., Buchanan A.S., Glass M. (2015). Pharmacology of Indole and Indazole Synthetic Cannabinoid Designer Drugs AB-FUBINACA, ADB-FUBINACA, AB-PINACA, ADB-PINACA, 5F-AB-PINACA, 5F-ADB-PINACA, ADBICA, and 5F-ADBICA. ACS Chem. Neurosci..

[11] Canazza I., Ossato A., Vincenzi F., Gregori A., Di Rosa F., Nigro F., Rimessi A., Pinton P., Varani K., Borea P.A., Marti M. (2017). Pharmaco-toxicological effects of the novel third-generation fluorinate synthetic cannabinoids, 5F-ADBINACA, AB-FUBINACA, and STS-135 in mice. In vitro and in vivo studies. Hum. Psychopharmacol..

[12] Schreiber S., Bader M., Lenchinski T., Meningher I., Rubovitch V., Katz Y., Cohen E., Gabet Y., Rotenberg M., Wolf E.U., Pick C.G. (2019). Functional effects of synthetic cannabinoids versus Δ 9 -THC in mice on body temperature, nociceptive threshold, anxiety, cognition, locomotor/exploratory parameters and depression. Addict. Biol..

[13] Trexler K.R., Vanegas S.O., Poklis J.L., Kinsey S.G. (2020). The short-acting synthetic cannabinoid AB-FUBINACA induces physical dependence in mice. Drug Alcohol Depend..

[14] Alzu’bi A., Abu-El-Rub E., Almahasneh F., Tahat L., Athamneh R.Y., Khasawneh R., Alzoubi H., Ghorab D.S., Almazari R., Zoubi M.S.A., Al-Zoubi R.M. (2024). Delineating the molecular mechanisms of hippocampal neurotoxicity induced by chronic administration of synthetic cannabinoid AB-FUBINACA in mice. Neurotoxicology.

[15] Carceles-Cordon M., Mannara F., Aguilar E., Castellanos A., Planagumà J., Dalmau J. (2020). NMDAR Antibodies Alter Dopamine Receptors and Cause Psychotic Behavior in Mice. Ann. Neurol..

[16] Tóth A., Petykó Z., Gálosi R., Szabó I., Karádi K., Feldmann Á., Péczely L., Kállai V., Karádi Z., Lénárd L. (2017). Neuronal coding of auditory sensorimotor gating in medial prefrontal cortex. Behav. Brain Res..

[17] Nishikawa M., Ito H., Tabata H., Ueda H., Nagata K.I. (2022). Impaired Function of PLEKHG2, a Rho-Guanine Nucleotide-Exchange Factor, Disrupts Corticogenesis in Neurodevelopmental Phenotypes. Cells.

[18] Stopponi S., Soverchia L., Ubaldi M., Cippitelli A., Serpelloni G., Ciccocioppo R. (2014). Chronic THC during adolescence increases the vulnerability to stress-induced relapse to heroin seeking in adult rats. Eur. Neuropsychopharmacol.

[19] Scherma M., Dessì C., Muntoni A.L., Lecca S., Satta V., Luchicchi A., Pistis M., Panlilio L.V., Fattore L., Goldberg S.R. (2016). Adolescent Δ 9-Tetrahydrocannabinol Exposure Alters WIN55,212-2 Self-Administration in Adult Rats. Neuropsychopharmacology.

[20] Izquierdo-Luengo C., Ten-Blanco M., Ponce-Renilla M., Perezzan R., Pereda-Pérez I., Berrendero F. (2023). Adolescent exposure to the Spice/K2 cannabinoid JWH-018 impairs sensorimotor gating and alters cortical perineuronal nets in a sex-dependent manner. Transl. Psychiatry.

[21] Saravia R., Ten-Blanco M., Julià-Hernández M., Gagliano H., Andero R., Armario A., Maldonado R., Berrendero F. (2019). Concomitant THC and stress adolescent exposure induces impaired fear extinction and related neurobiological changes in adulthood. Neuropharmacology.

[22] Rubino T., Realini N., Castiglioni C., Guidali C., Viganó D., Marras E., Petrosino S., Perletti G., Maccarrone M., Di Marzo V., Parolaro D. (2008). Role in anxiety behavior of the endocannabinoid system in the prefrontal cortex. Cereb. Cortex.

[23] Zuo Y., Iemolo A., Montilla-Perez P., Li H.R., Yang X., Telese F. (2022). Chronic adolescent exposure to cannabis in mice leads to sex-biased changes in gene expression networks across brain regions. Neuropsychopharmacology.

[24] Murphy M., Mills S., Winstone J., Leishman E., Wager-Miller J., Bradshaw H., Mackie K. (2017). Chronic Adolescent Δ9-Tetrahydrocannabinol Treatment of Male Mice Leads to Long-Term Cognitive and Behavioral Dysfunction, Which Are Prevented by Concurrent Cannabidiol Treatment. Cannabis Cannabinoid Res..

[25] Kevin R.C., Wood K.E., Stuart J., Mitchell A.J., Moir M., Banister S.D., Kassiou M., McGregor I.S. (2017). Acute and residual effects in adolescent rats resulting from exposure to the novel synthetic cannabinoids AB-PINACA and AB-FUBINACA. J. Psychopharmacol..

[26] Rubino T., Parolaro D. (2016). The impact of exposure to cannabinoids in adolescence: Insights from animal models. Biol. Psychiatry.

[27] Renard J., Krebs M.O., Jay T.M., Le Pen G. (2013). Long-term cognitive impairments induced by chronic cannabinoid exposure during adolescence in rats: A strain comparison. Psychopharmacology (Berl).

[28] Schneider M., Koch M. (2003). Chronic pubertal, but not adult chronic cannabinoid treatment impairs sensorimotor gating, recognition memory, and the performance in a progressive ratio task in adult rats. Neuropsychopharmacology.

[29] Rubino T., Parolaro D. (2011). Sexually dimorphic effects of cannabinoid compounds on emotion and cognition. Front. Behav. Neurosci..

[30] Gómez-Nieto R., Hormigo S., López D.E. (2020). Prepulse inhibition of the auditory startle reflex assessment as a hallmark of brainstem sensorimotor gating mechanisms. Brain Sci..

[31] Renard J., Vitalis T., Rame M., Krebs M.O., Lenkei Z., Le Pen G., Jay T.M. (2016). Chronic cannabinoid exposure during adolescence leads to long-term structural and functional changes in the prefrontal cortex. Eur. Neuropsychopharmacol.

[32] Renard J., Rosen L.G., Loureiro M., De Oliveira C., Schmid S., Rushlow W.J., Laviolette S.R. (2017). Adolescent Cannabinoid Exposure Induces a Persistent Sub-Cortical Hyper-Dopaminergic State and Associated Molecular Adaptations in the Prefrontal Cortex. Cereb. Cortex.

[33] Abela A.R., Rahbarnia A., Wood S., Lê A.D., Fletcher P.J. (2019). Adolescent exposure to Δ9-tetrahydrocannabinol delays acquisition of paired-associates learning in adulthood. Psychopharmacology (Berl).

[34] Garcia-Mompo C., Curto Y., Carceller H., Gilabert-Juan J., Rodriguez-Flores E., Guirado R., Nacher J. (2020). Δ-9-Tetrahydrocannabinol treatment during adolescence and alterations in the inhibitory networks of the adult prefrontal cortex in mice subjected to perinatal NMDA receptor antagonist injection and to postweaning social isolation. Transl. Psychiatry.

[35] Pintori N., Castelli M.P., Miliano C., Simola N., Fadda P., Fattore L., Scherma M., Ennas M.G., Mostallino R., Flore G. (2021). Repeated exposure to JWH-018 induces adaptive changes in the mesolimbic and mesocortical dopaminergic pathways, glial cells alterations, and behavioural correlates. Br. J. Pharmacol..

[36] Bilel S., Tirri M., Arfè R., Ossato A., Trapella C., Serpelloni G., Neri M., Fattore L., Marti M. (2020). Novel halogenated synthetic cannabinoids impair sensorimotor functions in mice. Neurotoxicology.

[37] Rubino T., Parolaro D. (2015). Sex-dependent vulnerability to Cannabis abuse in adolescence. Front. Psychiatry.

[38] Ruiz C.M., Torrens A., Castillo E., Perrone C.R., Cevallos J., Inshishian V.C., Harder E.V., Justeson D.N., Huestis M.A., Swarup V. (2021). Pharmacokinetic, behavioral, and brain activity effects of ?9-tetrahydrocannabinol in adolescent male and female rats. Neuropsychopharmacology.

[39] Nakano S., Nishikawa M., Kobayashi T., Harlin E.W., Ito T., Sato K., Sugiyama T., Yamakawa H., Nagase T., Ueda H. (2022). The Rho guanine nucleotide exchange factor PLEKHG1 is activated by interaction with and phosphorylation by Src family kinase member FYN. J. Biol. Chem..

[40] Edvardson S., Wang H., Dor T., Atawneh O., Yaacov B., Gartner J., Cinnamon Y., Chen S., Elpeleg O. (2016). Microcephaly-dystonia due to mutated PLEKHG2 with impaired actin polymerization. Neurogenetics.

[41] Rubino T., Realini N., Braida D., Guidi S., Capurro V., Viganò D., Guidali C., Pinter M., Sala M., Bartesaghi R., Parolaro D. (2009). Changes in hippocampal morphology and neuroplasticity induced by adolescent THC treatment are associated with cognitive impairment in adulthood. Hippocampus.

[42] Zhang Y., Wei C.K., Wang P., Zheng L.C., Cheng Y., Ren Z.H., Jin Y.H., Yao Y.Y., Liu H.Z. (2024). S-ketamine alleviates depression-like behavior and hippocampal neuroplasticity in the offspring of mice that experience prenatal stress. Sci. Rep..

[43] Yuan L., Song G., Xu W., Liu S., Zhang Y., Pan W., Ding X., Fu L., Lin Q., Sun F. (2024). Diethyl butylmalonate attenuates cognitive deficits and depression in 5×FAD mice. Front. Neurosci..

[44] Patro R., Duggal G., Love M.I., Irizarry R.A., Kingsford C. (2017). Salmon provides fast and bias-aware quantification of transcript expression. Nat. Methods.

[45] Love M.I., Huber W., Anders S. (2014). Moderated estimation of fold change and dispersion for RNA-seq data with DESeq2. Genome Biol..

[46] Zhong F., Liu L., Wei J.L., Dai R.P. (2019). Step by step golgi-cox staining for cryosection. Front. Neuroanat..

