# Supplementary material for: Long-term consequences of adolescent exposure to the synthetic cannabinoid AB-FUBINACA in male and female mice
Source: iScience. 2025 Jan 20;28(2):111857. doi: 10.1016/j.isci.2025.111857 (PMC11847088; doi:10.1016/j.isci.2025.111857)
Supplement: Document S1. Figures S1–S8 and Table S1 [file mmc1.pdf]

iScience, Volume *28*

Supplemental information

Long-term consequences of adolescent exposure to the synthetic cannabinoid AB-FUBINACA in male and female mice

Cristina Izquierdo-Luengo, María Ponce-Renilla, Marc Ten-Blanco, María Andrea Arnanz, Rosa María Tolón, Inmaculada Pereda-Pérez, and Fernando Berrendero

Assay	Parameters	Sex	Treatment effect	Interaction	N of animals	Normality	Equal variances	Figure	Statistical test
WEIGHT	Grams/day	Males	p = 0.1782	p **<0.0001**<br>$F_{(14, 1162)}$=10.79	54 per group	Yes	Yes	S1	ANOVA of RM
	AUC 1-5		p = 0.8472	-		Yes	Yes		T test
	AUC 6-10		p = 0.0848	-		Yes	Yes		T test
	AUC 11-15		p = **0.0114**	-		Yes	Yes		T test
	Grams/day	Females	p = 0.1304	p **<0.0001**<br>$F_{(14, 1372)}$=4.992	61 per group	Yes	Yes		ANOVA of RM
	AUC 1-5		p = 0.6559	-		Yes	No		T test Welch´s correction
	AUC 6-10		p = 0.1061	-		Yes	No		T test Welch´s correction
	AUC 11-15		p = **0.0003**	-		No	-		Mann-Whitney test
EPM	%Time OA	Males	p = **0.0142**	-	12 per group	Yes	No	1	T test Welch´s correction
	Total entries		p = 0.2708	-		Yes	Yes		T test
	%Time OA	Females	p = **0.0489**	-		Yes	Yes		T test
	Total entries		p = 0.5767	-		Yes	No		T test Welch´s correction
FC	% Freezing/cue	Males	p = 0.3794	p = 0.5869	12 per group	Yes	Yes	S3	ANOVA of RM
FE	% Freezing/session	Males	p = 0.8378	p = 0.6544		Yes	Yes	1	ANOVA of RM
FC	% Freezing/cue	Females	p = **0.0045**<br>$F_{(1, 20)}$ = 10.26	p = **0.0123**<br>$F_{(2, 40)}$ = 4.914	12 per group	Yes	Yes	S3	ANOVA of RM
FE	% Freezing/session	Females	p = 0.3262	p = 0.2794	12 per group	Yes	Yes	1	ANOVA of RM
LOC	AU	Males	p = 0.7450	-	13 per group	Yes	Yes	S2	T test
	AU	Females	p = 0.3411	-	14-15 per group	Yes	Yes		T test
NOR	Discrimination index	Males	p = 0.1286	-	14-15 per group	Yes	Yes	2	T test
	Total time exploring		p = 0.0927	-		Yes	Yes	S4	T test
	Discrimination index	Females	p = **0.0274**	-	11-13 per group	Yes	Yes	2	T test
	Total time exploring		p = 0.3134	-		Yes	Yes	S4	T test
SOC	Time direct contact/compartment	Males	p = 0.9942<br>*Compartment effect: <0.0001	p = 0.8034	14-16 per group	Yes	Yes	2	ANOVA
	Time direct contact/compartment	Females	p = 0.2777<br>*Compartment effect: <0.0001	p = 0.1826	14-15 per group	Yes	Yes		ANOVA
FST	Immobility time	Males	p = **0.0056**	-	14-15 per group	Yes	No		T test Welch´s correction
	Immobility time	Females	p = 0.7770	-		Yes	Yes		T test
PPI	% PPI	Males	p = 0.2515	p = 0.6083	13-15 per group	Yes	Yes	3	ANOVA of RM
	Mean % PPI		p = 0.2515	-		Yes	Yes		T test
	Startle response		p = 0.4442	-		Yes	Yes		T test
	% PPI	Females	p = **0.0210**<br>$F_{(1, 26)}$ = 6.040	p = 0.5177	13-15 per group	Yes	Yes		ANOVA of RM

	Mean % PPI		p = 0.0210	-		Yes	Yes		T test
	Startle response		p = 0.5070	-		Yes	Yes		T test
**PPI PND129**	% PPI	Females	p = 0.0856	p = 0.9107	14-15 per group	Yes	Yes	S5	ANOVA of RM
	Mean % PPI		p = 0.0856	-		Yes	Yes		T test
	Startle response		p = 0.2690	-		Yes	Yes		T test
**EPM Adults**	%Time OA	Females	p = 0.4515	-	10-14 per group	Yes	Yes	4	T test
	Total entries		p = 0.3836	-		Yes	Yes		T test
**NOR Adults**	Discrimination index	Females	p = 0.4483	-	13-14 per group	Yes	Yes		T test
	Total time exploring		p = 0.1385	-		Yes	Yes		T test
**PPI Adults**	% PPI	Females	p = 0.0329 $F_{(1, 27)} = 5.056$	p = 0.6999	12-17 per group	Yes	Yes		ANOVA of RM
	Mean % PPI		p = 0.0329	-		Yes	Yes		T test
	Startle response		p = 0.7730	-		Yes	Yes		T test
**Correlation PPI-RNAseq**	%PPI-Plekhg2	Females	p = 0.0007 r = -0.9336	-	4 per group	-	-	5	Pearson´s Correlation
	%PPI-Sh3tc1		p = 0.0171 r = -0.8003	-		-	-		Pearson´s Correlation
**Sholl Analysis**	Sholl	Females	p = 0.0181 $F_{(1,46)} = 6.01$	p <0.0001 $F_{(14, 370)} = 4.680$	6 neurons per mice and 4 mice per group	Yes	Yes	6	Mixed-ANOVA
	Length		p = 0.0286	-		No	-		Mann-Whitney test
	Convex hull		p = 0.0012	-		Yes	Yes		T test
	N of primary dendrites		p = 0.9408	-		Yes	No	S8	T test Welch´s correction
	N of secondary dendrites		p = 0.0265	-		Yes	Yes		T test
	N of tertiary dendrites		p = 0.0013	-		Yes	Yes		T test
**Dendritic spines analysis**	Total spine density (APICAL)	Females	p = 0.0064	-	4-5 neurons per mice and 4 mice per group	Yes	Yes	6	T test
	Long thin (APICAL)		p = 0.3143	-		Yes	No		T test Welch´s correction
	Stubby (APICAL)		p = 0.1143	-		No	-		Mann-Whitney test
	Mushroom (APICAL)		p = 0.0361	-		Yes	Yes		T test
	Branched (APICAL)		p = 0.3298	-		Yes	Yes		T test
	Total spine density (BASAL)		p = 0.8403	-		Yes	Yes	S8	T test
	Long thin (BASAL)		p = 0.5636	-		Yes	Yes		T test
	Stubby (BASAL)		p = 0.8307	-		Yes	Yes		T test
	Mushroom (BASAL)		p = 0.3810	-		No	-		Mann-Whitney test
	Branched (BASAL)		p = 0.7749	-		Yes	Yes		T test
**CDC42 G-LISA**	Relative activity	Females	p = 0.8208	-	11 per group	Yes	Yes	S7	T test

Table S1: statistical summary of all experiments are shown. P value is considered significant (blue) under 0.05. F values are shown when significant effect was found after ANOVA analysis. AUC area under the curve, AU arbitrary units, EPM elevated plus maze, FC fear conditioning, FE fear extinction, FST forced swimming test, LOC locomotion, N number, NOR novel object recognition, OA open arms, PND post-natal day, PPI prepulse inhibition, RM repeated measures, SOC sociability.

Figure S1

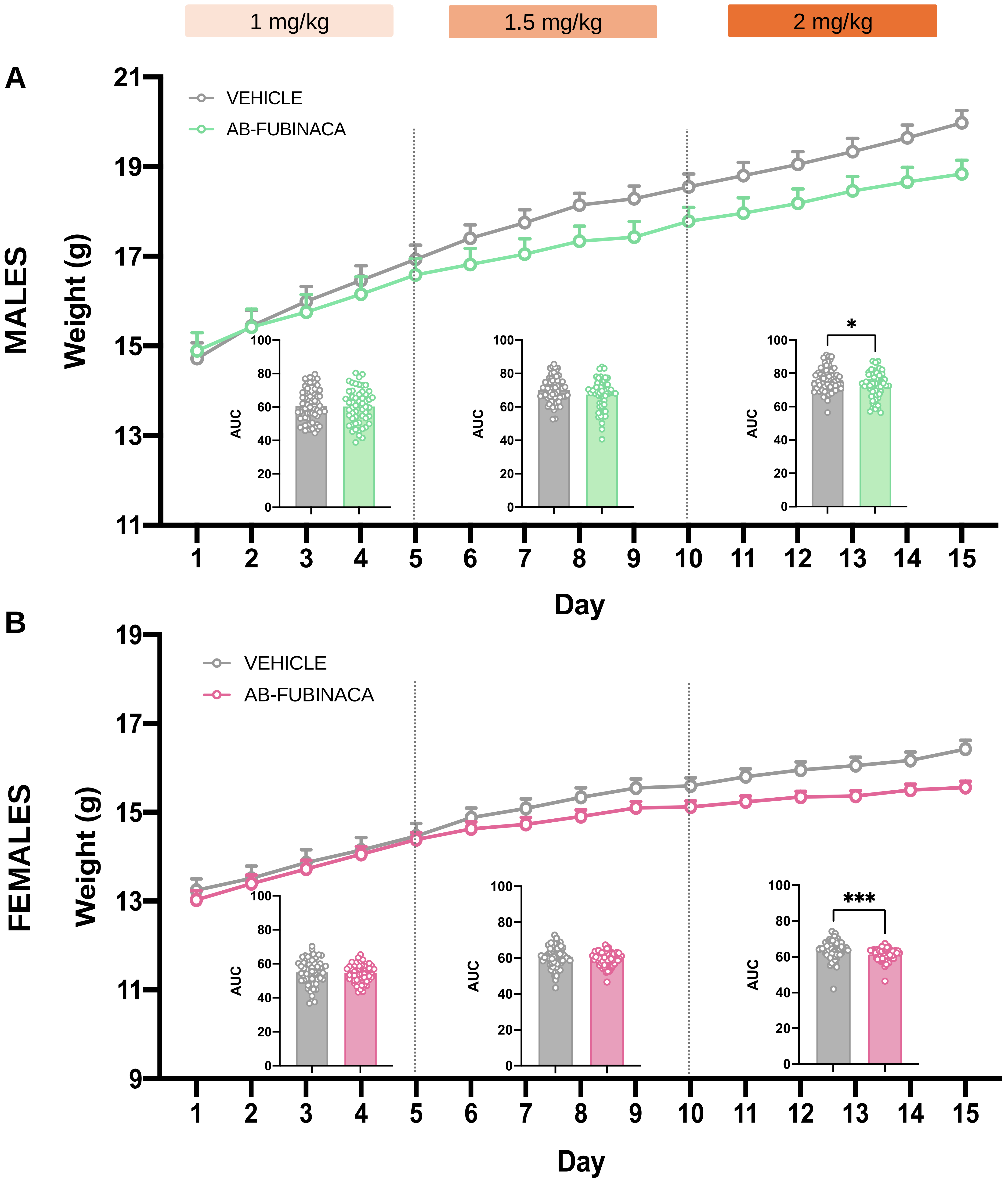

Fig. S1. Adolescent exposure to AB-FUBINACA alters body weight in male and female mice. Effects of treatment with AB-FUBINACA during adolescence in body weight of adolescent male (**A**) and female (**B**) mice (n= 54-61 mice per group). Daily weight in grams during the 15 days of treatment and AUC values every five days are shown. Data are expressed as mean ± SEM. *p < 0.05, ***p < 0.001 (comparison between AB-FUBINACA and vehicle; Student's t-test (**A, B**)). AUC, area under the curve.

Figure S2

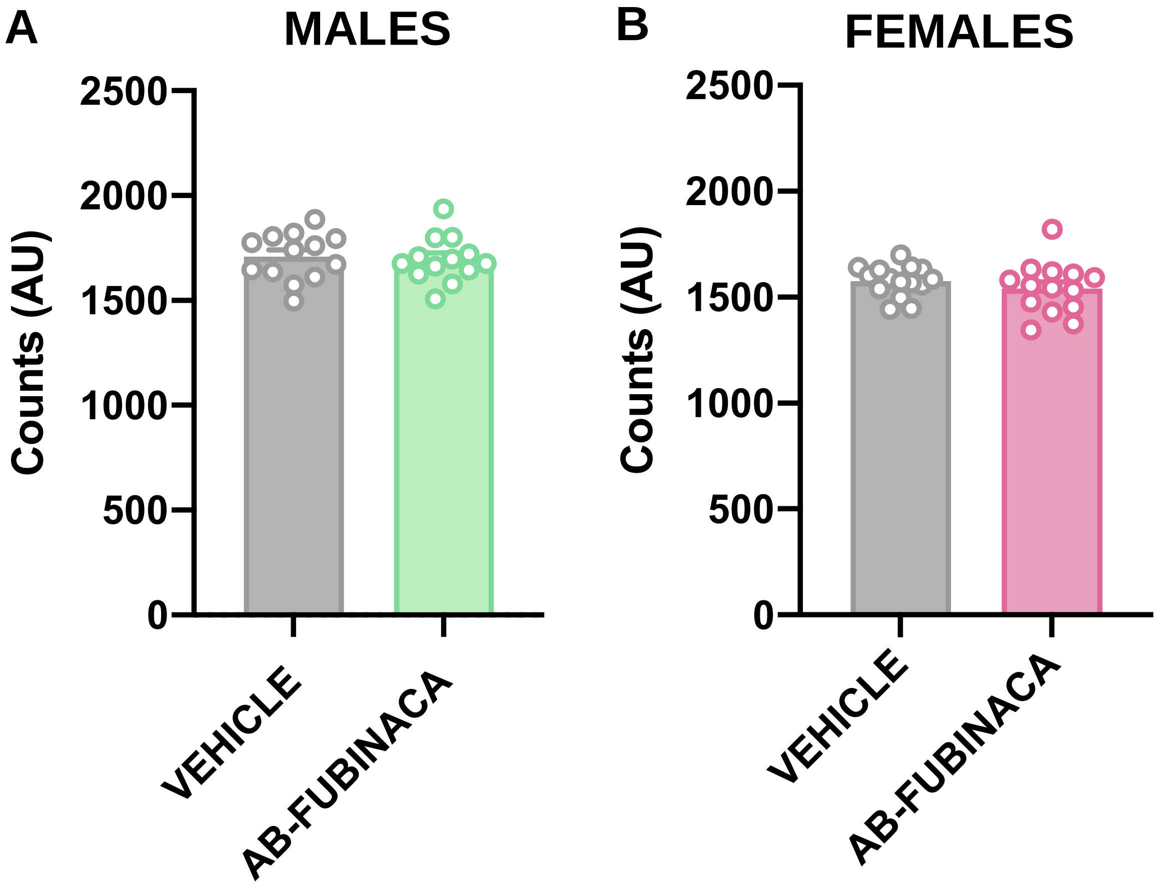

Fig. S2. Adolescent exposure to AB-FUBINACA does not modify locomotion in male and female mice. Effects of treatment with AB-FUBINACA during adolescence on locomotor activity in adult male (**A**) and female (**B**) mice (n = 13-15 mice per group). Data are expressed as mean ± SEM. AU, arbitrary units.

Figure S3

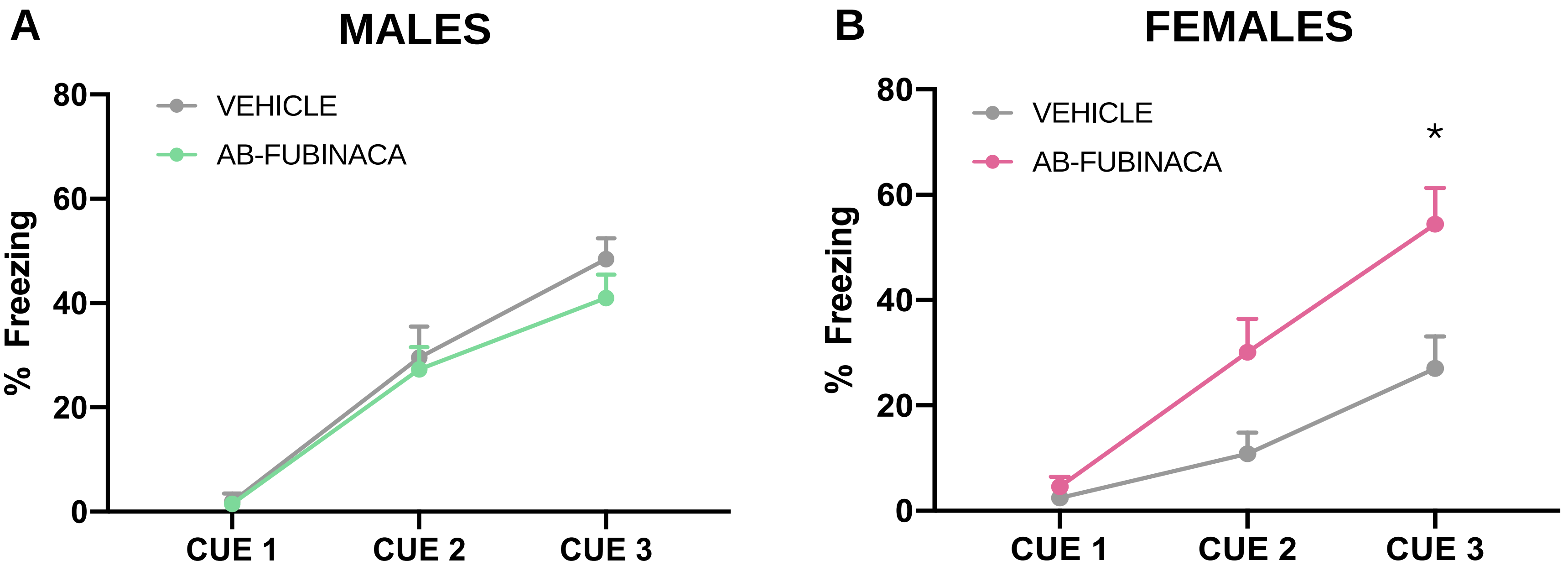

Fig. S3. Adolescent exposure to AB-FUBINACA alters fear conditioning in female mice. Effects of treatment with AB-FUBINACA during adolescence in fear conditioning in adult male (**A**) and female (**B**) mice (n = 12 mice per group). Time course of the freezing levels scored during each cue is shown for fear conditioning. Data are expressed as mean ± SEM. *$p < 0.05$ (comparison between AB-FUBINACA and vehicle group; two-way ANOVA with repeated measures (**B**)).

Figure S4

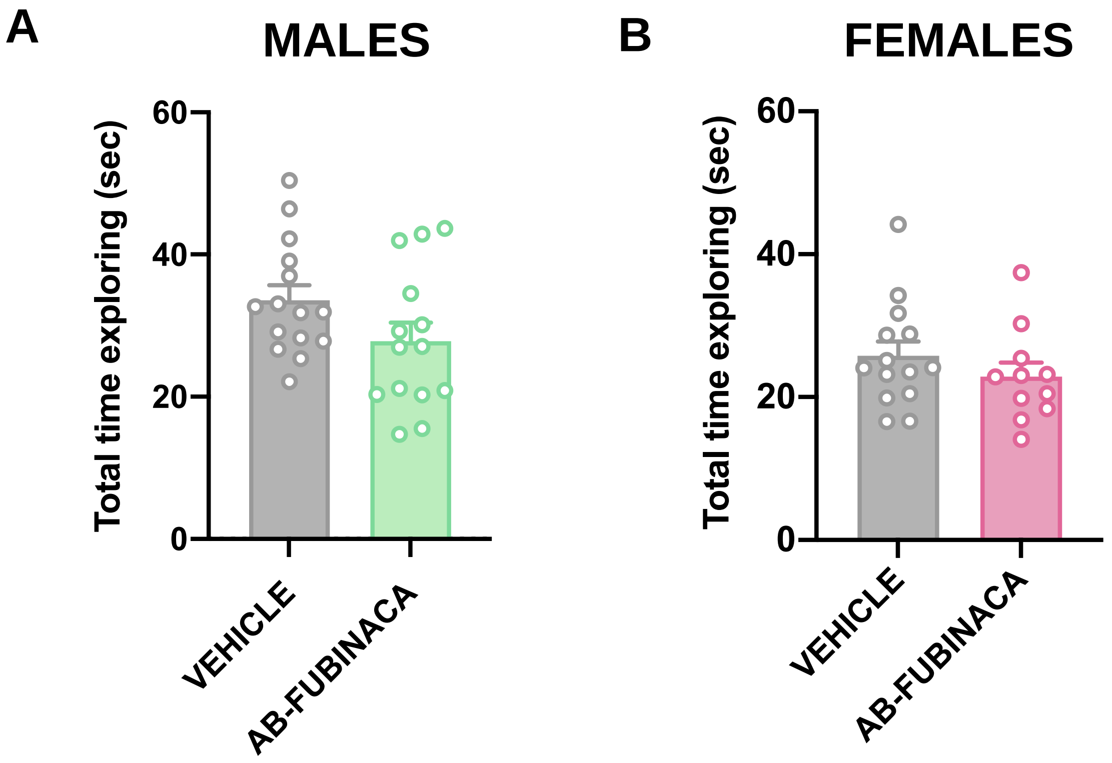

Fig. S4. Adolescent exposure to AB-FUBINACA does not alter total time of exploration in the novel object recognition test in males or females. Effects of treatment with AB-FUBINACA during adolescence on time of exploration in the NOR in adult male (**A**) and female (**B**) mice (n = 11-15 mice per group). Data are expressed as mean ± SEM. Sec, seconds.

Figure S5

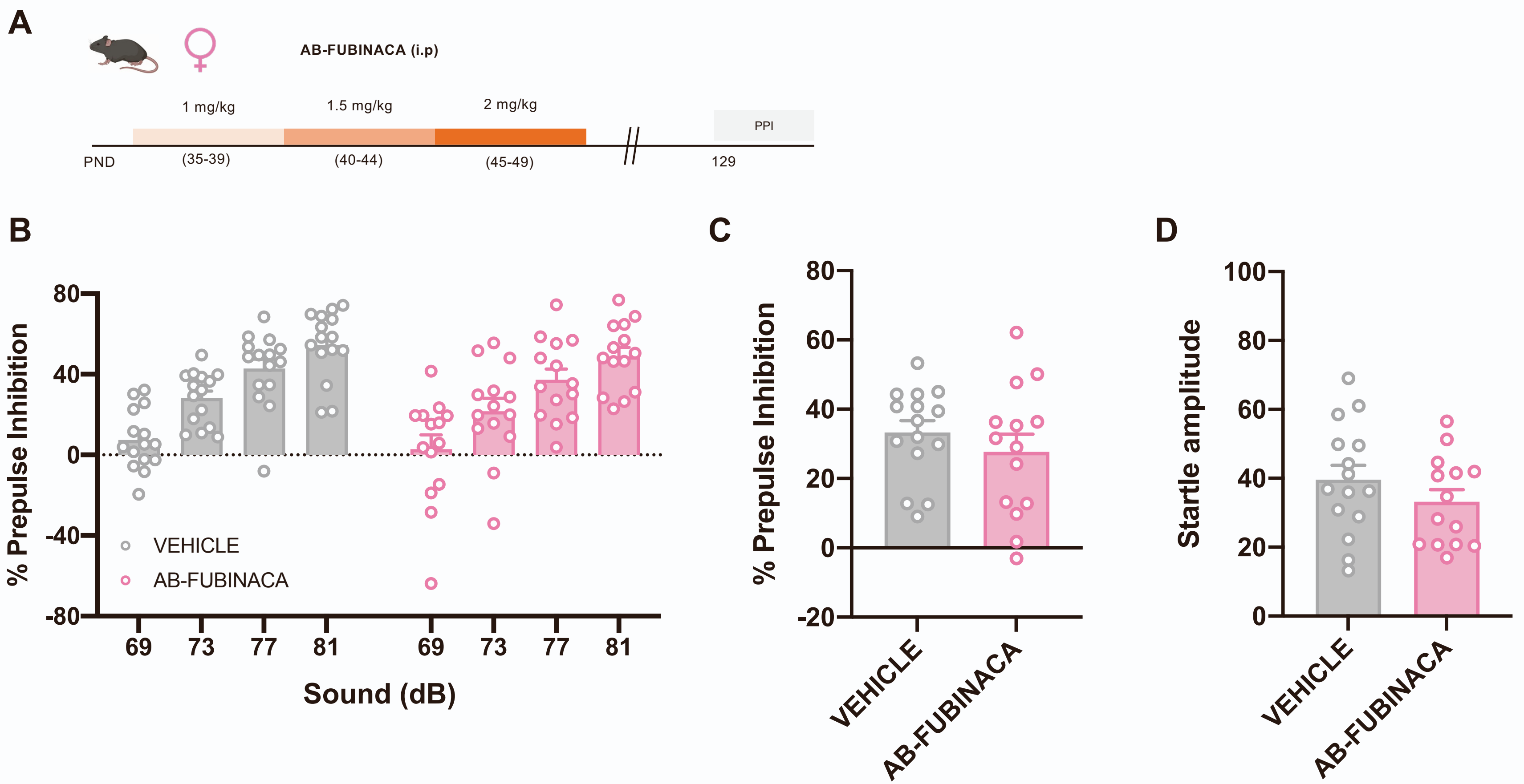

Fig. S5. Adolescent exposure to AB-FUBINACA does not alter sensorimotor gating 80 days after the end of the treatment in female mice. (**A**) Schematic representation of the experimental design. (**B-D**) Effects of treatment with AB-FUBINACA during adolescence 80 days after the last day of treatment on PPI in adult female mice (n = 14-15 mice per group). Percentage of prepulse inhibition (**B**), mean of the percentage of prepulse inhibition (**C**), and startle response amplitude (**D**) are shown. Data are expressed as mean ± SEM. PND, postnatal day, PPI prepulse inhibition test.

Figure S6

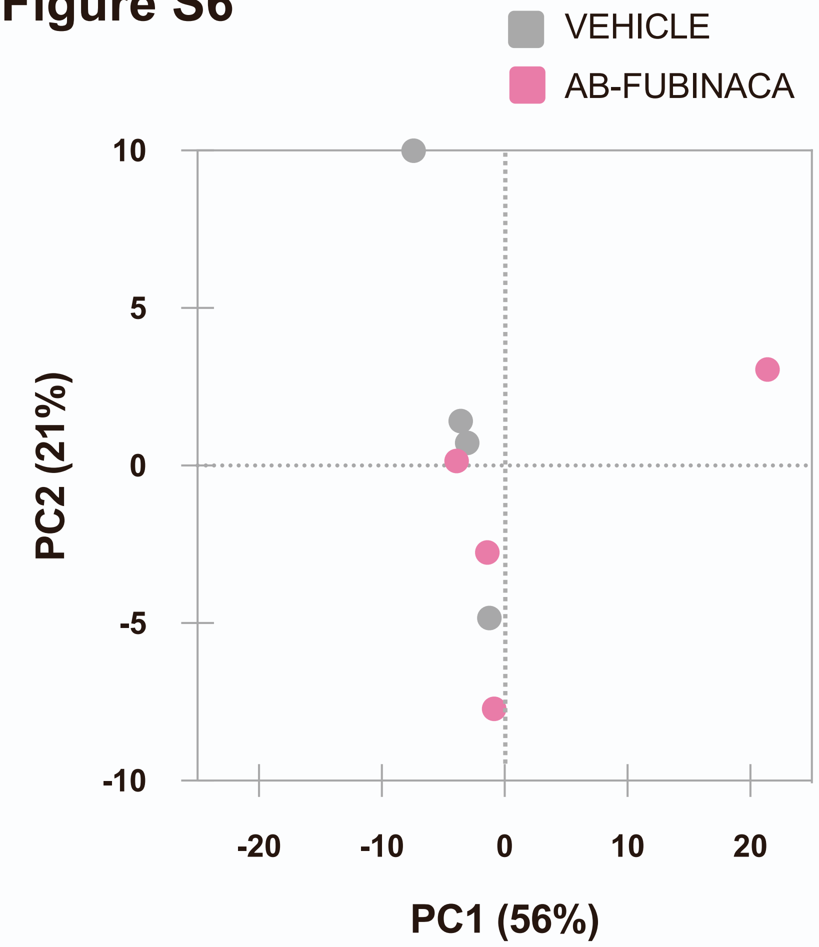

Fig. S6. Principal component analysis using the expression patterns of genes in the prefrontal cortex in adult female mice treated with AB-FUBINACA or vehicle during adolescence. (n = 4 mice per group). PC, principal components 1 and 2.

Figure S7

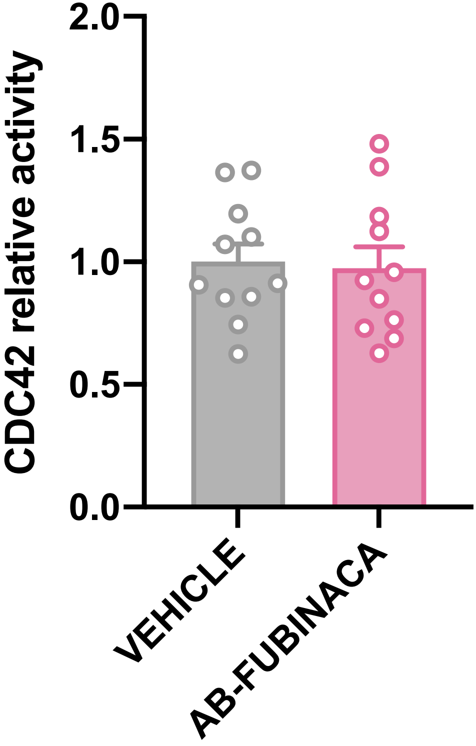

Fig. S7. Adolescent exposure to AB-FUBINACA does not modify CDC42 activity in the prefrontal cortex of adult female mice. (n = 11 mice per group)

Figure S8

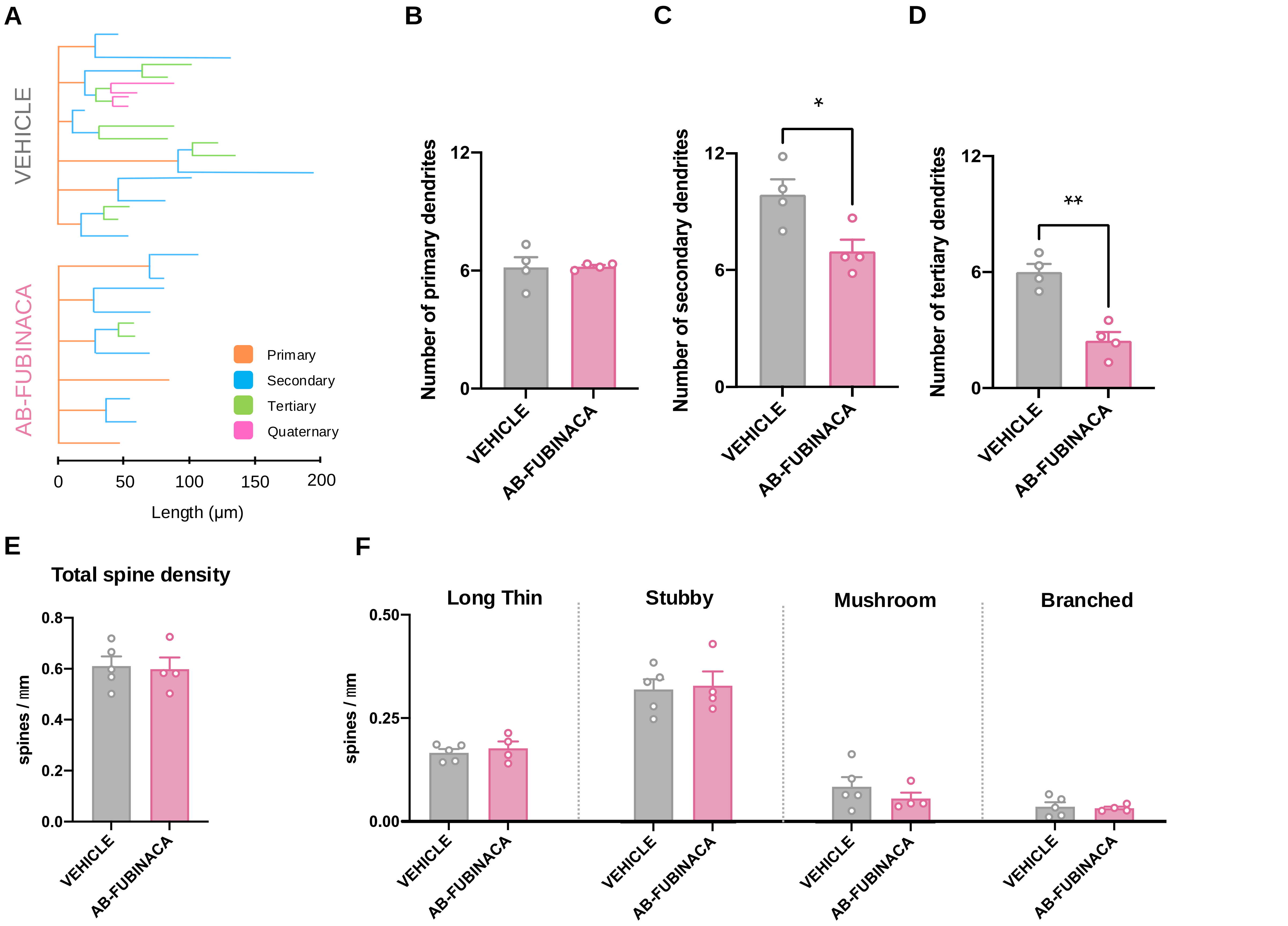

Fig. S8. Adolescent exposure to AB-FUBINACA induces alterations in dendritic complexity, but not in spine density in basal dendritic spines, in the prefrontal cortex of adult female mice. (**A**) Representative dendrogram of neurons of the prefrontal cortex in AB-FUBINACA or vehicle treated female mice. Dendrogram is colored by branch order. (**B-D**) Number of dendrites classified by orders in adult females treated with AB-FUBINACA or vehicle during adolescnece (n = 6 neurons/animal, n = 4 mice per group). Primary (**B**), secondary (**C**) and tertiary dendrite orders (**D**) are shown. (**E**) Total spine density of basal dendrites in neurons of the prefrontal cortex between in adult females treated with AB-FUBINACA or vehicle during adolescence (n = 4-5 neurons/animal, n = 4 mice per group). (**F**) Spine density grouped according to their morphological characteristics. Data are expressed as mean ± SEM. *p < 0.05 and **p < 0.01 (comparison between AB-FUBINACA and vehicle group; Student's t-test (**C, D**)).
